# Computer interface for a diode-array based spectrophotometer

**DOI:** 10.1155/S1463924683000280

**Published:** 1983

**Authors:** Harry L. Pardue, Steven D. Hamilton, William E. Weiser, Harris Kirk, Ioannis Laois

**Affiliations:** 1 Department of Chemistry Purdue University West Lafayette Indiana 47907 USA; 2 E. R. Squibb PO Box 4000 Princeton New Jersey 08540 USA


Journal of Automatic Chemistry, Volume 5, Number 2 (April–June 1983), pages 108–110

### Computer interface for a diode-array based spectrophotometer

**Harry L. Pardue, Steven D. Hamilton, William E. Weiser, Harris Kirk* and Ioannis Laois**
*Department of Chemistry, Purdue University, West Lafayette, Indiana 47907, USA*

### Introduction

One of the significant developments in ultraviolet/visible (UV/VIS) spectrophotometry in recent years involves the adaptation of array detectors to monitor multiple wavelengths simultaneously [1]. The rapid-scanning capability of these devices simplifies many measurement and data-processing options (derivative spectroscopy, multicomponent quantitation, multiwavelength kinetic studies etc.) that are more difficult to implement with conventional, mechanically scanned systems [2]. To fully exploit these and other options, it is necessary to use these imaging detectors in conjunction with on-line computers.

The Hewlett-Packard 8450A UV/VIS spectrophotometer utilizes two photodiode arrays to permit the optical spectrum between 200 and 800 nm to be scanned with a repetition rate of one scan per second. Although the spectrophotometer includes a 16-bit computer with a wide variety of data-processing options, it is probable that, like us, many users will need data-processing capabilities that are not included in the instrument. Therefore, it is judged that an interface to a general-purpose computer for which an extensive array of programs is available would be of general interest. This paper describes an interface for the HP8450A UV/VIS spectrophotometer designed specifically for Hewlett-Packard 2100 computers; it is also compatible with the 1000 Series of computers from the same manufacturer. Data for an illustrative application are discussed briefly.

### Description of interface

This presentation is limited to a description of the system; complete details of hardware and software will be provided upon request.

### *Hardware*

All the control functions needed to operate the spectrophotometer and manipulate raw data are provided by a 16-bit microcomputer with a 28k ROM operating system and 32k RAM storage. A link to external devices is provided by a Hewlett-Packard Interface Bus (HPIB) port and a RS-232C bit-serial interface. The HPIB, the Hewlett-Packard version of the IEEE-488 standard, is used to interface standard Hewlett-Packard accessories such as digital plotters and cassette-storage units. The RS-232C port is used to interface the spectrophotometer to external computers.

The HP2100A computer used in this application is configured with 32k of core memory and operated with the DOS-M operating system. Computer peripherals include a CRT display, a line-printer, a dual 8 in floppy disc drive for system control and data storage, and a Tektronics 603 storage oscilloscope for rapid display of data.

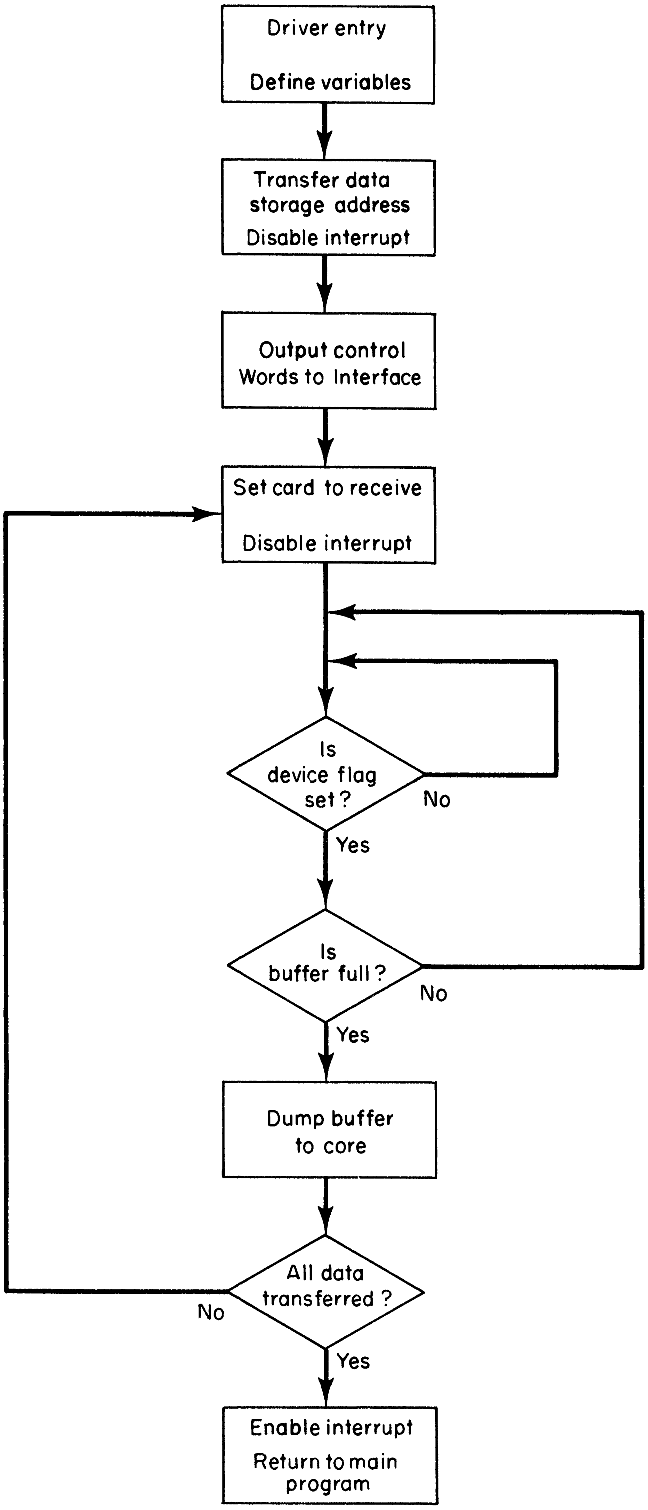

*Figure 1. Flow chart of assembly language driver.*

*Present address: E. R. Squibb, PO Box 4000, Princeton, New Jersey 08540, USA.

The heart of the interface is a Hewlett-Packard 12966A Buffered Asynchronous Data Communications Interface Card. This card has a wide range of applications because it is compatible with both the Hewlett-Packard 2100 and 1000 Series of computers. Pertinent features of the card include hardwired or software-selectable baud rates, software-selectable parity and parity sense, character or buffer mode, character length, stop bits, and several interrupt options. This application uses the buffer mode in which 128 8-bit bytes are transferred to the buffer before being transmitted to the spectrophotometer or computer.

### *Software*

The HP8450A data-file structure consists of 832 16-bit words of data. The first 30 words of data represent a header which describes the type of data (for example wavelength versus absorbance, absorbance versus time etc.) and the number of active data words. The remaining 802 words are the packed floating point binary data. All 832 words of data are transferred each time, regardless of the number of active words contained in the data file.

The interface driver routine is written in assembly language as a non-interrupt driver. The driver is called by a single statement in Fortran IV with the data stored in a COMMON block. The driver operates as illustrated in the flow chart in figure 1. After control words are output to the interface, the control bit is set on the interface card. After receiving this signal, the spectrophotometer transfers data to the interface buffer until the buffer status word indicates the buffer is full. The RTS line is set low and the buffer content is transferred into core. This process is repeated until all 832 words are transferred. In the receive mode (spectrophotometer to computer) there is handshaking on both ends of the line, so that either device will wait for the other to be ready. However, in the transmit mode (computer to spectrophotometer) the spectrophotometer can receive only binary data and so it must be readied before control is set on the interface. The spectrophotometer does not routinely poll the RS-232C interface for data being sent; if the computer attempts to send data when the spectrophotometer is not ready to receive, the data will be lost and the spectrophotometer keyboard will be disabled.

### User programs

Two FORTRAN IV data-transfer programs have been developed to be compatible with the assembly language driver routine. One program is designed for bidirectional data transfer

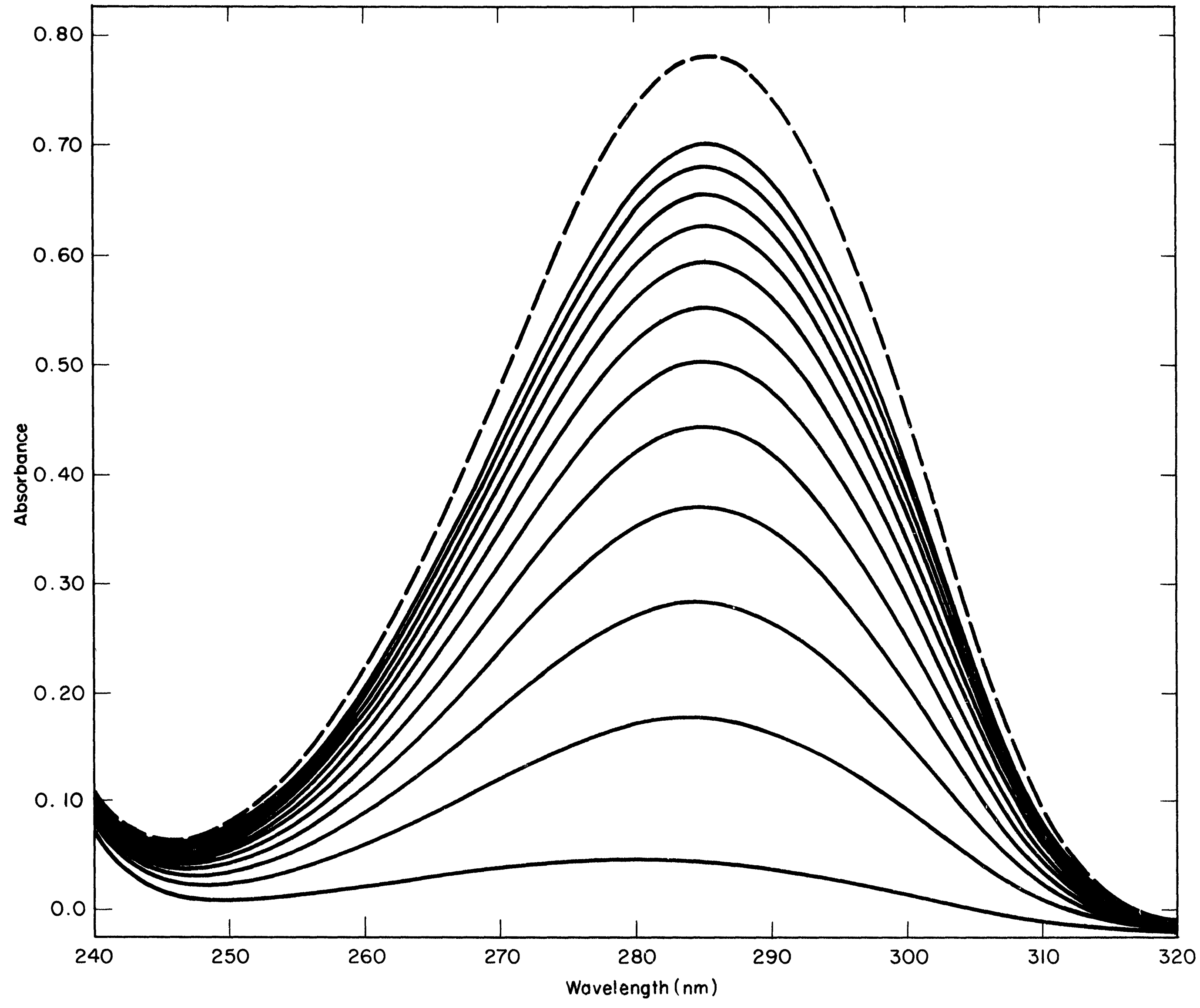

*Figure 2. Reaction of acetoacetate and glycine monitored between 240 and 320 nm. Time between scans is 200s. The dotted curve is the equilibrium spectrum taken at 6000s.*

and data listing. The user is presented with a choice or seven program options. These options are:

(1) Transfer of a single HP8450A data file into computer memory.
(2) Transfer of multiple HP8450A data files onto sequential disc files.
(3) Transfer of data file from memory to disc.
(4) Transfer of data file from disc to memory.
(5) List data file in memory.
(6) Transfer of memory data file to HP8450A.
(7) Program exit.

Any choice can be made at the beginning of the program and again after each operation. Data transferred to the computer can originate as HP8450A display data, stored standards, or tape files. Cassette tapes (Hewlett-Packard 200) are used for short-term data storage during high instrument-demand periods; tape files can be transferred in bulk to disc storage (option 2 above) during low demand periods. The listing option provides a CRT or hard-copy list of all or part of a data file, along with the descriptive information in the data file header.

The second program mentioned above is designed to extract absorbance versus time data for up to five wavelengths from absorbance versus wavelength measurements made at timed ($> 3 = $s) intervals. Absorbance versus time files for each wavelength chosen are created with a 30-word header to make them identical to spectrophotometer generated data files. These data files are then stored on floppy disc and displayed on the oscilloscope.

The absorbance versus time data can be processed in any desired manner. A typical example includes a multipurpose nonlinear regression program. This program includes a subroutine that converts the HP845OA format data, including the data contained in the 30-word header (scan rate, dead time, and number of data points) into standard FORTRAN IV floating point format. Program options permit the data to be fit to a zero-order model, a first-order model [3], a mixed zero- and first-order model [4] or a Michaelis–Menten model [5]. A batch-mode option allows as many as 30 data files to be processed without user interaction.

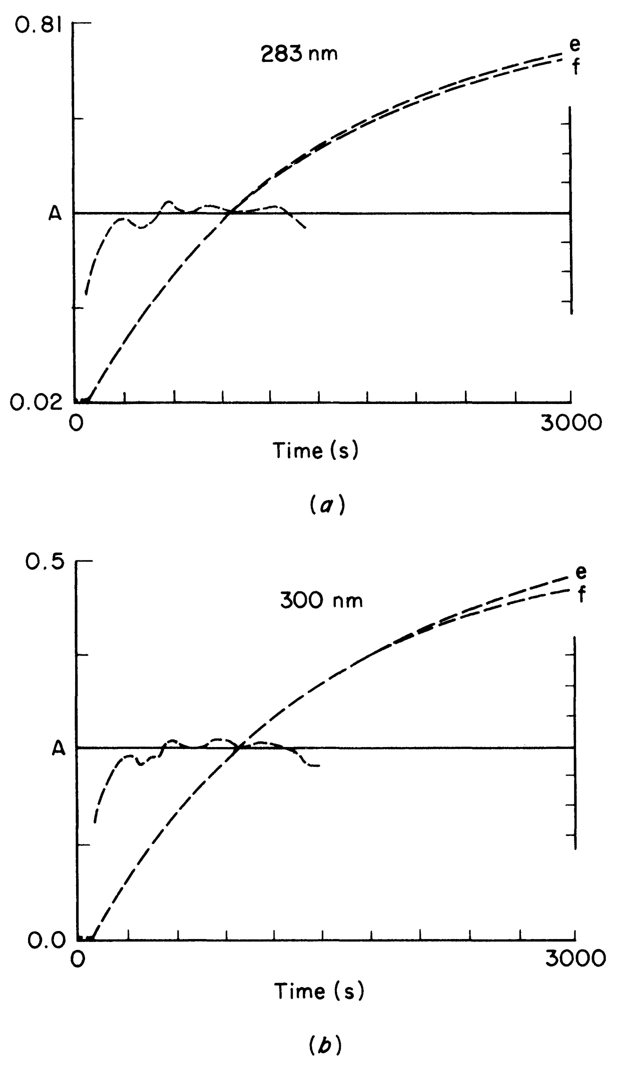

*Figure 3 (a and b). Experimental (e) and computed (f) response curves, with residuals (horizontal axis) scaled to the left absorbance axis. Data rate—50s per scan. Fitting range—1450s.*

### Example application

The HP8450A spectrophotometer has been used with the interface hardware and software to study the reaction of acetoacetate with glycine at a pH of 8·6 with a $Na_2HPO_4$ buffer at 298 K. Absorbance versus time data monitored between 240 and 320 nm are illustrated in figure 2. Typically, the computer interface is used to extract in real-time absorbance versus time data for five wavelengths. These data files are then processed using the multipurpose nonlinear regression program described above.

Figures 3(*a*) and 3(*b*) show some absorbance versus time data for the reaction at 283 nm and 300 nm. Each figure includes both experimental data and results obtained by fitting the experimental data to a first-order model over a range of 1·5 half-lives (1 – 1450 s). Residuals of the fit are plotted on the horizontal axis near the midpoint of the ordinate. The primary purpose in this study was to predict the equilibrium absorbance change without monitoring the reaction to completion [3]. Alternately, one could compare rate constants computed at the different wavelengths to determine if there are any complicating factors such as absorbance of intermediates or unexpected products. In this case, rate constants evaluated at 260, 284, and 300 nm were 5·9, 7·1, and 6·6 $s^{-1} \times 10^{-4}$, respectively. This and other applications will be discussed in more detail in later publications.

In summary, with a modest cost of time and money, this interface has permitted us to use this advanced spectrophotometer with a wide range of software that has been developed during the past decade.

### Acknowledgement

This work was supported in part by Grant No. GM 13326-15 from the National Institutes of Health, USA.

### References

1. Talmi, Y., in *Multichannel Image Detectors*, Ed. Talmi, Y., No. 102, ACS Symposium Series (American Chemical Society, 1979).
2. Pardue, H. L., in *Topics in Automatic Chemistry*, Ed. Forman, J. K. and Stockwell, P. B. (Horwood, Chichester, 1978), p. 163.
3. Meiling, G. E. and Pardue, H. L., *Analytical Chemistry*, **25** (1978), 1611.
4. Harner, R. S. and Pardue, H. L., *Analytica Chimica Acta*, **127** (1981), 23.
5. Hamilton, S. D. and Pardue, H. L., *Clinical Chemistry* (forthcoming).

